# A Case Study of Intractable Vomiting with Final Diagnosis of Neuromyelitis Optica

**DOI:** 10.1155/2015/291390

**Published:** 2015-10-05

**Authors:** Rachel Bramson, Angela Hairrell

**Affiliations:** ^1^College of Medicine, Texas A&M Health Science Center College of Medicine, Bryan, TX 77807, USA; ^2^Family Medicine, Scott and White University Clinic, College Station, TX 77845, USA

## Abstract

This case study presents a patient living in a suburban/rural community who received appropriate referral to secondary and tertiary care for nausea and vomiting, accompanied by waxing and waning neurological symptoms, yet proved difficult to diagnose. This patient is presented to draw attention to a rare neurological disorder which should be included in the differential diagnosis of nausea and vomiting with some key neurological complaints, even in the absence of physical findings.

## 1. Introduction

When a previously healthy adolescent presents with nausea and vomiting, the most common diagnoses are viral gastroenteritis or pyelonephritis. Vague waxing and waning neurological symptoms in an adolescent may be attributed to stress or developmental issues. Here we present a patient living in a suburban/rural community who received appropriate referral to secondary and tertiary care, yet proved difficult to diagnose. This patient is presented to draw attention to a rare neurological disorder which should be included in the differential diagnosis of nausea and vomiting with some key neurological complaints, even in the absence of physical findings. Physical findings may develop late in the course of the disease making a heightened index of suspicion important for early diagnosis and treatment.

## 2. Patient Presentation

The patient is a 17-year-old African-American female, multisport athlete in 11th grade in a rural high school. She is the second of four children and lives at home with her parents and two siblings. She gets Bs and Cs in school and is extremely well-liked by teachers and fellow students. She presented to Urgent Care with abdominal pain, nausea, and vomiting with a 19 lb. unintentional weight loss over a three-month period and BMI of 20. Her past medical history revealed normal development, immunizations being up to date, and pyelonephritis at age 14 for which she was hospitalized. She had no past surgeries and no history of alcohol, tobacco, or drug use. Her parents and siblings are healthy.

## 3. Clinical Findings

On initial presentation, the patient appeared healthy with no notable physical findings. She was discharged on Zofran and a clear liquid diet. On a follow-up two days later she had mild epigastric tenderness on physical exam and described a problem with shaky hands and back pain radiating into the right leg off and on, worse when she runs. The neurological exam was normal. CBC and CMP were normal with the exception of low WBC 3400 and sodium 134. TSH was low normal (0.5). On Beck's Anxiety Inventory she scored 11 (mild), and on Beck's Depression Inventory she scored 1 (negative).

## 4. Timeline

Due to the protracted course of this patient's illness and workup, Table 1 details her visits and care over a three-month period. The following is a brief summary of her clinical presentation.

Two days after her initial presentation, she presented to the emergency room with vomiting and dehydration. Bloodwork and chest X-ray were unremarkable except for low prealbumin. She was treated with IV fluids and discharged on promethazine to relieve the nausea and vomiting. Three days later, she presented with intractable vomiting and a five-day history of hiccoughs. She was admitted to the local hospital and transferred the same day to the regional children's hospital. She was discharged eight days later with a biopsy-proven diagnosis of* H. pylori* gastritis and hypertension having had multiple imaging studies, upper and lower endoscopy, and renal and nutrition consultation for 15% weight loss in three months. Neurological exams revealed normal reflexes, strength, tone, coordination, and movement. Discharge medication included triple therapy for* H. pylori*, sucralfate, clonidine patch, and ondansetron.

Six days later, she presented to the emergency room and was transferred back to the regional children's hospital for pancreatitis and hypertension with additional history of fleeting blurry vision and pruritus of the left face. Workup included a neurology consult, brain imaging, EGD with biopsy, and echocardiogram, all unremarkable. Neuropathic facial pain was added to her diagnoses. Treatment continued unchanged.

In follow-up office visit two days later, the patient's mother insisted she was having lower extremity weakness, shaking episodes that looked like seizures, and hypersensitivity of the face and head. An urgent referral to neurology resulted in hospitalization at the regional children's hospital. Neurological exam showed slightly decreased strength of the left upper extremity and bilateral lower extremities, pronator drift, and gait weakness on the left side. Video EEG, EMG, and nerve conduction studies were unrevealing. Wilson's disease was ruled out. The patient was found to have a vitamin D deficiency, normal cortisol, and negative ANA.

Nine days later in follow-up with the primary care provider, tremors were witnessed and the patient had intense pain of the bilateral upper extremities, chest, and low back. Urgent referral to pediatric neurology resulted in an order for MRI of the whole spine with and without contrast which was performed eight days later. The next day, the radiology results were described to the patient (increased signal in the medulla oblongata, between C2 and C5, and possible diagnosis of syringomyelia). Three days later she saw a pediatric neurosurgeon who suspected a multilevel spinal cord tumor and referred her to a tertiary care children's hospital department of neurosurgery. Due to difficulty scheduling an outpatient consultation, the patient presented to the emergency room nine days later and was admitted for a possible multilevel spinal cord tumor.

## 5. Outcome

At the tertiary care children's hospital, CSF from lumbar puncture was NMO positive with 18 WBC, 3 RBC, gram stain and culture negative, IgG index 0.6, oligoclonal bands negative, ACE 1.1, and NMDA receptor antibody negative. MRI of the cervical spine revealed confluent ill-defined, partly enhancing, abnormal intramedullary signal from the pontomedullary junction through the lower cervical cord with no optic nerve abnormality (no syringomyelia or spinal cord tumor). She was also diagnosed with extensive cervical myelopathy and dystonia. She was started on carbamazepine, two days of IV IG, and five days of IV methylprednisone and discharged on a prednisone taper.

Eight days later she followed up with pediatric neurology and started mycophenolate with the goal of a 1000 mg BID. She was noted to have Secondary Paroxysmal Kinesigenic Dyskinesia (PKD).

Six weeks later in a follow-up with her primary care physician, she had gained 34 pounds and was doing well on mycophenolate, carbamazepine, and prednisone.

## 6. Patient Perspective

Mother's perspective: I knew something was wrong with my daughter. It was frustrating to have to ask for testing. I am glad that we finally got a diagnosis. Now my daughter is much better.

## 7. Discussion

Physicians involved in the management of this patient were diligent in efforts to reveal the underlying cause of intractable nausea and vomiting. Several factors contributed to the difficulty of determining the correct underlying diagnosis. (1) Nausea and vomiting were the presenting problem for one month. (2) The patient's neurological symptoms presented late and were vague and diffuse with only subtle findings on physical exam. While her mother reported “shaking spells,” the tremors observed appeared to be a stress response. (3) The normal CT and MRI of the brain were falsely reassuring that a demyelinating disorder or other neurological disease was not the underlying cause. (4) The patient developed neck pain late in the course of her illness. The combination of tremors, paresthesias, nonspecific muscle tenderness, and weakness led to whole spine MRI. The cervical spine MRI revealed the abnormal findings which allowed subsequent diagnosis.

This patient's diagnostic workup also demonstrates a critical point about interpretation of abnormal spinal cord images: a false diagnosis of spinal cord tumor can result in unnecessary biopsy of the spinal cord. Fortunately, the neurosurgeon referred the patient to a tertiary care setting where further evaluation resulted in the correct diagnosis and treatment.

Neuromyelitis optica (NMO) is an uncommon disease syndrome of the central nervous system (CNS) that affects the optic nerves and spinal cord. Individuals with NMO develop optic neuritis (pain in the eye and vision loss) and transverse myelitis (weakness, numbness, and sometimes paralysis of the arms and legs), along with sensory disturbances and loss of bladder and bowel control [1–5].

NMO is distinguished from multiple sclerosis by positive serum autoantibody NMO-IgG, which targets aquaporin 4 [6–11]. Over the last nine years, the aquaporin 4 serum test has allowed identification of a wider spectrum of clinical and radiological characteristics associated with NMO.

In 2006 [3], diagnostic criteria were developed for a related clinical syndrome, NMOSD (NMO Spectrum Disorder). This requires NMO-IgG seropositive status in association with a limited form of NMO or a “signature clinical syndrome,” such as intractable nausea, vomiting, or hiccoughs [5, 12, 13].

This patient demonstrates several typical findings of NMO, presented here to encourage clinicians to identify this unusual constellation of symptoms. NMO should be considered in the differential for intractable nausea and vomiting [13]. Analysis of a multicenter study by Mealy et al. [14] provides the best current characteristics of patients with NMO and NMOSD. This study examined 187 patients who were diagnosed with NMO or NMODS at three nationally known medical centers distributed across the US.

Our patient shares several characteristics with patients in the multicenter study. As in other autoimmune disorders, females predominate (6.5 : 1) [14]. African Americans were overrepresented at 37% [14], while they represent only 13.2% of the US population [15]. Additionally, of those patients in the study with brain stem lesions (*n* = 30/187), 80% (*n* = 24/30) were of African descent [14]. The disproportionate prevalence of NMO in African Americans and their increased incidence of brain stem lesions deserve further study. However, while our patient was only 17, the median age of onset in the study group was 40 years (range: 3–81) [14].

Other common manifestations of NMO were also noted in this patient [5]. For example, most patients with transverse myelitis due to NMO have extensive longitudinal involvement (≥3 vertebral segments), as did our patient. Additionally there are reported cases of generalized pruritus early in the disease [9, 16, 17]; this patient had focal pruritus of the left cheek which became hyperpigmented due to inflammation and scratching. This was the only clue to the itching she was experiencing. This patient also developed hypertension.

NMO has yet to receive funding for a national multicenter consortium; however, a five-year multicenter analysis in 2012 [14] revealed several key features of the disease that this patient demonstrates. Pediatricians and other primary care providers should be sensitized to the unusual presenting symptoms of NMO to optimize early detection and treatment. Early detection and treatment seem to improve prognosis and reduce relapse rate.

## Figures and Tables

**Table 1:** Timeline of presentation and diagnosis.

Date	Chief complaint/history	Consultant	Diagnostic study	Findings and treatment
*9/7/2014* Urgent care visit	(i) Vomiting (ii) Unintentional weight loss	Primary care		Prescribing ondansetron and follow-up with PCP for weight loss

*9/9/2014* PCP follow-up office visit	(i) Gastritis (ii) Weight loss (iii) Orthostatic hypotension (iv) Intermittent right lower back pain with radiation into the right leg	Primary care	(i) Beck's Anxiety and Depression Inventory (ii) CBC, CMP (iii) Lipid Panel (iv) TSH	(i) Mild anxiety (ii) No depression (iii) All labs normal (iv) Normal reflexes, muscle tone, coordination

*9/11/2014* ER visit	(i) Vomiting (ii) Dehydration (iii) Weight loss		(i) Chest X-ray (ii) CBC, CMP (iii) Urine pregnancy test and UA (iv) ESR (v) CRP	(i) All normal, except low prealbumin at 18 and sodium 136 (ii) Given IV fluids (iii) Discharged on promethazine for vomiting and nausea

*9/14–9/22/2014* Community hospital admittance with same day transfer to children's hospital	(i) Intractable vomiting (ii) Weight loss (iii) Hypertension (iv) 5-day history of hiccoughs	Pediatric GI	Right upper quadrant ultrasound	Small amount of gall bladder sludge, no gallstones
			CT abdomen pelvis with contrast	Normal
			EGD	(i) *H*. *Pylori* gastritis, biopsy diagnosis (ii) Triple therapy was initiated, changed to IV due to persistent nausea and vomiting, completed
			Colonoscopy with biopsies	No abnormalities
		Pediatric nephrology	Urine VMA, Renal ultrasound	Both normal
		Pediatric nutrition		(i) 15% weight loss in 3 months (ii) 27th percentile weight for age (iii) 32nd percentile stature for age
		Neurology		Normal strength, tone, movement
		*Discharge Diagnosis*: *H*. *pylori* gastritis *medication*: Triple therapy for *H*. *pylori*: clonidine patch, pantoprazole, sucralfate		

*9/28–10/4/2014* ER visit/transfer to children's hospital	(i) Pancreatitis (ii) Hypertension *History*: 2 fleeting episodes of blurry vision, lightheadedness, pain, itching on left side of face since 9/24, syncope on 9/25 with 10-second loss of consciousness, difficulty following commands	Pediatric neurology	(i) Brain CT (ii) Brain MRI	(i) Both Normal (ii) Normal strength, tone, movement (iii) PT consult for instability in ambulation
		Pediatric GI	EGD with biopsy for emesis	(i) Gastric nodularity, *H*. *pylori* positive (ii) Lipase 465–1498, Lactic acid 2.7 elevated
		Pediatric nephrology	Echo	Normal
		*Discharge diagnosis*: Dehydration, abdominal pain, intractable vomiting, syncope, *H*. *pylori* infection, neuropathic left facial pain (worsened on gabapentin, so stopped) *Medication*: Clonidine patch 0.1 mg/24 hr weekly, pantoprazole 40 mg 2x daily, sucralfate 1 g 2x daily prn, ondansetron 8 mg 2x daily prn		

*10/6/2014* PCP follow-up from hospitalization	(i) Pancreatitis (ii) Hypertension *History*: lower extremity weakness, numbness in fingers, hyperesthesia of the face and head, shaking episodes, feeling cold, mom reports symptoms worsening, had “seizure” in hospital		(i) Lipase (ii) West Nile IGG and IGM (iii) ESR (iv) TSH	(i) Physical exam: normal reflexes, no cranial nerve deficits, normal muscle tone, decreased sensation in extremities (ii) Labs normal except elevated lipase (277) (iii) Cold intolerance (iv) Spells (v) Urgent referral to neurology

*10/7–9/2014* Office visit with pediatric neurology resulting in hospitalization at children's hospital	*History*: shaking episodes since 9/27 affecting head and upper extremities, occur randomly, last seconds with no postictal confusion, pins and needles sensation affecting entire face and left arm, itching and burning of left face with hyperpigmentation, weakness requiring wheelchair	Pediatric neurology, pediatric GI, adolescent medicine, neurosurgery	Neurological exam	(i) Cranial nerves intact (ii) Sensation intact (iii) Bilateral upper and lower extremities (iv) Right upper extremity strength 5/5 (v) Left upper extremity strength 4/5 (vi) Pronator drift (vii) Bilateral lower extremities 4/5 (viii) Gait weak on left side
			Continuous 24-hr. Video EEG	Normal in awake, drowsy, sleeping states; 7 episodes with no EEG correlate
			EMG and Nerve conduction study	Normal
			(i) Ceruloplasmin (ii) Vitamin D (iii) Cortisol (iv) Lipase (v) ANA profile (vi) Entrovirus	(i) Low (ii) Low (iii) Normal (iv) Elevated lipase (771) (v) Negative (vi) Negative
		*Discharge Diagnosis*: (i) Left-sided upper and lower extremity weakness (ii) Spells *Medications*: Vitamin D deficiency, hydrocortisone cream, ondansetron, sucralfate, vitamin D, multivitamin, ranitidine		

*10/18/2014* PCP f/u hospitalization	(i) Pain in bilateral upper extremities, chest, low back (ii) Shaking		Lipase	(i) Almost normal (126) (ii) Myalgias (iii) Parasthesias (iv) Spells (v) Tremors (vi) Hypertension (vii) Urgent referral to pediatric neurology

*10/23/2014* Pediatric neurology	(i) Pain in bilateral upper extremities, chest, low back (ii) Shaking	Pediatric neurology	(i) MRI whole spine, with and without contrast (performed on 11/3) (ii) Neurological exam	(i) Tremors (ii) Parasthesias (face, arms, trunk, legs) (iii) Nonspecific muscle tenderness, possible weakness (iv) Reflexes normal, downgoing plantar Muscle normal bulk, tone, strength (v) Decreased cold face to T4, decreased pinprick face to toes. (vi) Recent history of pancreatitis

*10/29/2014* pediatric GI f/u hospitalization	Intractable vomiting	Pediatric GI	Serum copper	Normal

*11/4/2014* PCP follow-up	MRI results		(i) MRI cervical spine (ii) MRI thoracic spine, lumbar spine, sacrum, coccyx	(i) 4 × 3 cystic dilation of the central canal from C5 to T2 (ii) Increased signal in the medulla oblongata and C2 to C5, relative expansion of the cervical spinal cord (iii) Syringomyelia (iv) Normal

*11/7/2014* pediatric neurosurgery	Abnormal cervical spine MRI *History*: decreased activity due to neck pain, difficulty starting urination, hand tremor, weakness in all extremities, numbness and tingling in left leg	Pediatric neurosurgery	Neurological exam	(i) Abnormal reflexes, increased muscle tone, clonus in both feet, spastic gait (ii) No muscle atrophy, no cranial nerve or sensory deficit, normal position and vibration sense, normal coordination (iii) Possible multilevel spinal cord tumor (iv) Referred to 2nd children's hospital neurosurgery

*11/16–25/2014* ER visit and hospitalization at a second children's hospital	Possible multilevel spinal cord tumor	Child neurology	(i) Lumbar puncture (ii) MRI cerv. spine	(i) CSF positive for neuromyelitis optica (NMO), WBC 18, RBC 3, gram stain culture negative, IgG index 0.6, oligoclonal bands negative, ACE 1.1, NMDA receptor antibody negative; seropositive for NMO. (ii) Extensive cervical spine longitudinal myelitis; confluent ill-defined, partly enhancing, abnormal intramedullary signal from pontomedullary junction through the lower cervical cord. No optic nerve abnormality. (iii) Dystonia (iv) Tegretol 300 mg 3x daily (v) 2 days of IV IG (vi) 5-day course of IV methylprednisone, discharged on prednisone taper
		*Discharge diagnosis*: (i) Neuromyelitis optica (NMO) (ii) Cervical myelopathy (iii) Dystonia		

*12/3/2014* Follow-up with pediatric neurology				(i) Neuromyelitis optica (NMO) (ii) Cervical myelopathy (iii) Secondary Paroxysmal Kinesigenic Dyskinesia (PKD) (iv) Start Cellcept with slow increase to goal of 1000 mg BID

*1/22/2015* Follow-up with PCP	(i) Neuromyelitis optica (ii) Cervical myelopathy			(i) Weight gain since November of 34 lb (ii) Taking mycophenolate, carbamazepine, prednisone (iii) Referral to ophthalmology for baseline evaluation and ongoing care
